# DNA Sequence Determinants Controlling Affinity, Stability and Shape of DNA Complexes Bound by the Nucleoid Protein Fis

**DOI:** 10.1371/journal.pone.0150189

**Published:** 2016-03-09

**Authors:** Stephen P. Hancock, Stefano Stella, Duilio Cascio, Reid C. Johnson

**Affiliations:** 1 Department of Biological Chemistry, David Geffen School of Medicine at the University of California at Los Angeles, Los Angeles, California, United States of America; 2 Department of Energy Institute of Genomics and Proteomics, University of California at Los Angeles, Los Angeles, California, United States of America; 3 Molecular Biology Institute, University of California at Los Angeles, Los Angeles, California, United States of America; Florida International University Bimolecular Sciences Institute, UNITED STATES

## Abstract

The abundant Fis nucleoid protein selectively binds poorly related DNA sequences with high affinities to regulate diverse DNA reactions. Fis binds DNA primarily through DNA backbone contacts and selects target sites by reading conformational properties of DNA sequences, most prominently intrinsic minor groove widths. High-affinity binding requires Fis-stabilized DNA conformational changes that vary depending on DNA sequence. In order to better understand the molecular basis for high affinity site recognition, we analyzed the effects of DNA sequence within and flanking the core Fis binding site on binding affinity and DNA structure. X-ray crystal structures of Fis-DNA complexes containing variable sequences in the noncontacted center of the binding site or variations within the major groove interfaces show that the DNA can adapt to the Fis dimer surface asymmetrically. We show that the presence and position of pyrimidine-purine base steps within the major groove interfaces affect both local DNA bending and minor groove compression to modulate affinities and lifetimes of Fis-DNA complexes. Sequences flanking the core binding site also modulate complex affinities, lifetimes, and the degree of local and global Fis-induced DNA bending. In particular, a G immediately upstream of the 15 bp core sequence inhibits binding and bending, and A-tracts within the flanking base pairs increase both complex lifetimes and global DNA curvatures. Taken together, our observations support a revised DNA motif specifying high-affinity Fis binding and highlight the range of conformations that Fis-bound DNA can adopt. The affinities and DNA conformations of individual Fis-DNA complexes are likely to be tailored to their context-specific biological functions.

## Introduction

A large number of diverse DNA transactions require the cooperative formation of higher-order nucleoprotein complexes including transcription, replication, DNA repair, and DNA segregation. An in depth understanding of the sequence-dependent variability in protein-induced DNA structure is necessary to understand the molecular mechanisms that direct the formation of these nucleoprotein complexes. In this work we study the bacterial nucleoid-associated protein, Fis, and its association with a variety of DNA sites in order to investigate the role of DNA sequence in target selection and protein-stabilized DNA shape changes within the bound complexes.

Fis is one of a handful of nucleoid-associated proteins that bind prolifically throughout bacterial chromosomes. It is among the most abundant DNA binding proteins under rapid growth conditions in Enterobacteriaceae. Over 1000 binding peaks, many of which contain multiple binding sites, have been identified in genome-wide studies in *Escherichia coli* and *Salmonella enterica* [1–3]. Fis regulates transcription, recombination, and replication reactions and is implicated in chromosome packaging [4–8]. Transcriptome analyses have shown that Fis directly or indirectly modulates RNA levels of over 20% of chromosomal genes, including many that are involved in protein translation, metabolite transport, and virulence functions [1–3,9,10]. Fis functions as a primary gene regulator by binding to specific DNA sites and recruiting RNA polymerase through cooperative interactions with the alpha or sigma subunits [11–13]. Fis also cooperatively recruits or competes with alternative regulators or effector proteins including recombinases. In several of these reactions, Fis-induced changes in DNA shape has been shown to contribute to the formation of higher-order nucleoprotein complexes [5,14–16].

**Fig 1:**
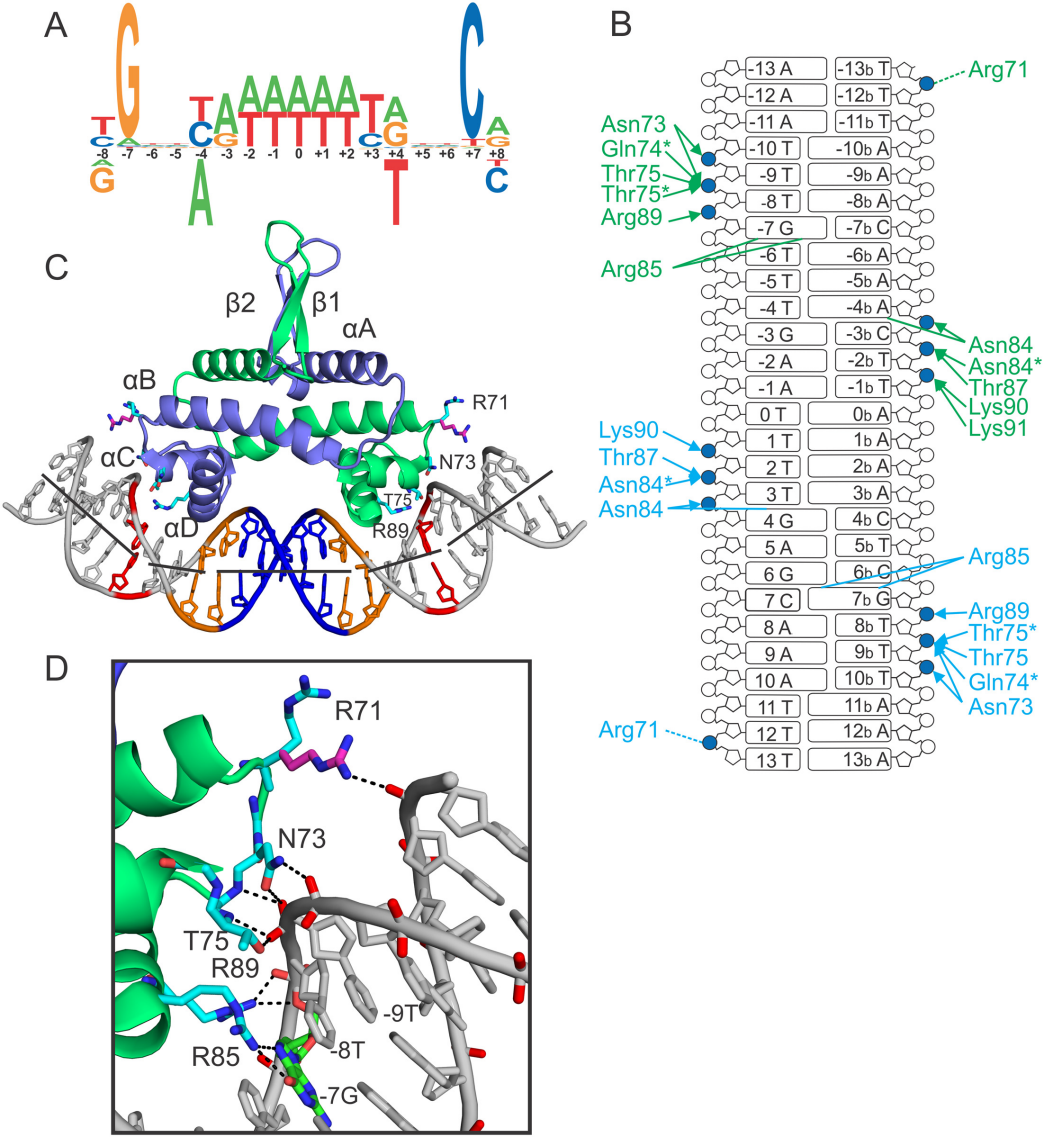
Fis binding site logo and contacts made to the Fis-bound DNA. **(A)** Qualitative sequence logo generated from a compilation of well-defined Fis binding sites, many of which have been shown to function directly as regulatory sites in transcription or recombination reactions, mutagenesis studies [29], and X-ray crystal structures (this paper and [28–30]). The core motif is defined here as sequences between -7 and +7, and flanking sequences extend beyond these limits. Bases depicted below the numbering inhibit binding. This symmetric motif differs from recent genome-wide chromatin immunoprecipitation studies where motif finders return binding logos that exhibit asymmetrically positioned A-tracts within the core [1,2]. **(B)** Ladder diagram of Fis-DNA contacts in the reference Fis-F1 crystal structure (PDB ID: 3IV5). DNA phosphates are shown as circles, ribose sugars as pentagons, and bases as rectangles. Phosphates that are contacted are filled blue. Contacts made by chain A side chains (light blue) and those made by chain B (green) are shown. Asterisks represent contacts made by protein backbone atoms. Arrows represent contacts made to the phosphate backbone, whereas lines represent contacts to the bases. **(C)** Crystal structure of the high affinity Fis-F1 DNA complex. Secondary structural elements are labeled and shown as a cartoon. Fis side chains that contact the flanking DNA backbone are shown as sticks. Sequence elements that are hallmarks of high affinity Fis binding sites including the conserved ±7(G/C) (red), the ±(3–4) Y-R step (orange), and the A/T rich center (blue). Lines through the helical axis have been drawn to highlight the points of helix axis deflection. **(D)** Zoomed-in representation of the residues that contact the flanking DNA backbone. The Arg71 side chain, for which experimental electron density is weak, has been modeled in purple to interact with the ±(12–13) phosphate here and in panel C (see also [22].

**Fig 2:**
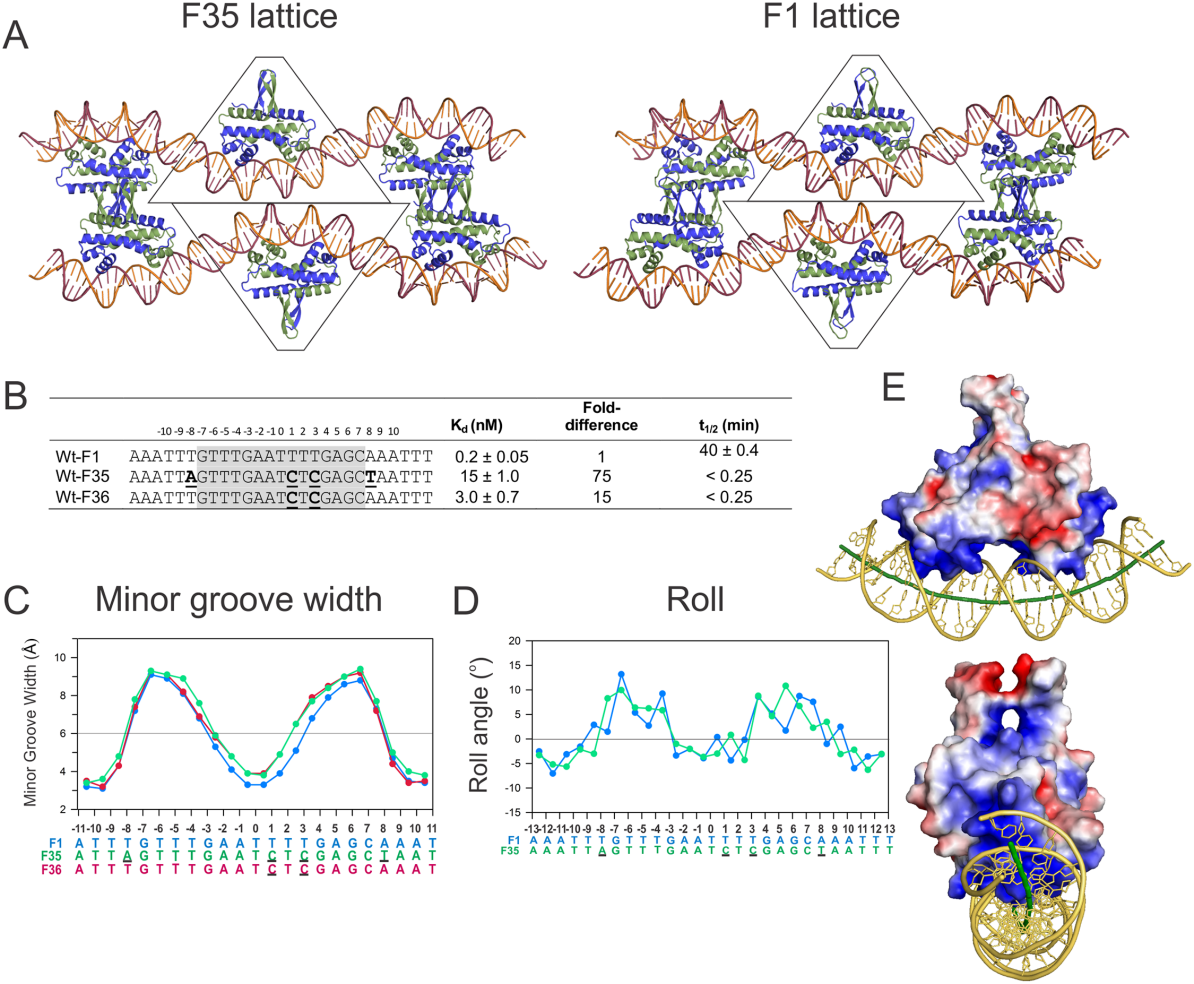
Effects of the F35 base substitutions on Fis-DNA binding and structure. **(A)** Crystal lattice differences between the F35 (left) and F1 (right) complexes. The contents of one asymmetric unit are bound by a trapezoid. Protein chains A and B are represented as blue and green cartoons, respectively, and DNA chains C and D are shown as magenta and orange cartoons. The relationship between symmetry mates is reversed in F35 as compared to F1. **(B**) Binding affinities and lifetimes for the F36 and F35 sequences relative to F1. The 15 bp core sequences are highlighted in grey. **(C)** Plot of minor groove width (van der Waal’s radii subtracted) for the bound DNAs in the F35, F36, and F1 complexes.) The color of the plots corresponds to the color of the DNA sites below. Bases that are different than those in F1 are bold and underlined. **(D)** Roll angle plots of the bound DNAs in the F35 relative to F1 complexes. **(E)** Electrostatic potential calculations (±3 kT/e) mapped on to the surface of the F35 Fis molecule. Blue and red colors represent electropositive and electronegative surfaces, respectively. Note the DNA writhe as it follows the basic track extending along the sides of the Fis dimer. The surface potential map and DNA track of the F35 complex is indistinguishable from the F1 complex.

In vitro, Fis promiscuously and dynamically binds DNA [17,18], but forms long lived complexes at specific sites with subnanomolar to nanomolar affinities [19]. The sequences of high-affinity Fis binding sites are remarkably diverse but have been described as 15 bp AT-rich core elements bounded by G/C and C/G base pairs. Fig 1A shows a sequence logo that incorporates an extensive list of well-characterized high-affinity Fis binding sites, genome-wide binding data, mutagenesis, and structural studies. In addition to the conserved G/C base pairs at the boundaries and the A/T-rich center, a pyrimidine-purine (Y-R) step is present at the ±(3–4) positions in ~80% of half-sites of well-characterized regulatory Fis sites. This feature was recognized in early compilations of stable Fis binding sites [19–23] but is not present in more recent consensus sequence studies, including those utilizing motif finders on genome-wide ChIP data [1,2]. Y-R steps exhibit low base stacking, and as a consequence, are often sites of helix bending [24–27] as is observed at the ±(3–4) position in X-ray crystal structures of Fis bound to high affinity sites (Figs 1C and 2D) [28].

Multiple X-ray crystal structures of Fis-DNA complexes reveal that most of the Fis contacts are to the DNA backbone and extend over a 21 bp segment (Fig 1B–1D) [28]. The conserved guanines at ±7 are directly contacted by the side chain of Arg85, the first residue of the recognition α-helices that protrude into the major groove (Fig 1D). The Asn84 side chain contacts a purine at -4b/+4 when present (Fig 1B); mutagenesis studies show that the Asn84-purine contact is much less important for binding than Arg85, but the Asn84 side chain strongly discriminates against the presence of a thymine at -4b/+4 because of a clash with the C5 methyl group [28,29]. The importance of the A/T-rich center (-2 to +2) is due to the requirement for a compressed minor groove, which enables the recognition helices in the Fis dimer to contact the consecutive major grooves [28,30]. The DNA is nearly straight over the central 5 bp but is curved towards the Fis protein over the major groove interfaces (Fig 1C). In complexes formed on optimal Fis binding sequences, positive roll angle peaks at the ±(6–7) and the ±(3–4) Y-R steps, deflect the DNA axis and enable the Arg85-G7 and phosphate backbone contacts at the edges of the DNA interface (Fig 1C and 1D). The Fis-bound DNA typically exhibit overall curvatures of ~65–70°, but crystal lattice interactions likely impact the trajectories of the flanking DNA segments.

High affinity regulatory Fis binding sites tend to be A/T-rich outside of the 15 bp core with a T/A overrepresented at the ±8 position (Fig 1A) [1,2,22,23]. All the Fis-DNA X-ray structures show a set of five hydrogen bonds between protein side chains or backbone amides and DNA phosphates extending out to ±10 (Fig 1B). These contacts clamp the Fis dimer onto the duplex and stabilize the curved DNA structure. The minor groove over the flanking regions is also significantly narrowed and faces the basic sides of the Fis surface, consistent with hydroxyl radical cleavage protection over these regions [31,32]. Biochemical studies have provided evidence that Arg71 strongly influences wrapping of the DNA toward the sides of Fis [22,33]. The Arg71 side chains are not well resolved in the X-ray data but are believed to dynamically contact the backbone between ±(12–13) [22].

Here we investigate the effects of DNA sequence within the major groove interface and flanking the 15 bp core on the affinity, stability, and structure of high affinity Fis-DNA complexes. We present six new Fis-DNA crystal structures that provide detailed information illustrating how sequence variations influence the way that DNA conforms to the Fis binding surface. One of the complexes crystallized in a space group that has not previously been observed. We illustrate how DNA sequence affects Fis-bound DNA conformation in the three structurally distinct regions: the central narrow minor groove, the kinked major grooves, and the curved flanking segments.

## Results

### Structure of a Fis-DNA complex in a different crystal form

During the course of Fis-DNA co-crystallization trials, we obtained a 2.9 Å resolution structure of the Fis-F35 complex that crystallized with C2 symmetry, which differs from the P2_1_2_1_2_1_ space group observed for all other reported Fis-DNA crystal structures (this paper and [28,30]). The F35 sequence differs from our canonical high affinity F1 binding site at four positions: +1C, and +3C in the core and -8A and +8T in the flanks (Fig 2B). The lattice organization of the F35 crystals resembles that of F1 in that the DNA adopts a serpentine pseudo-continuous helix; however, the Fis dimer subunits are oriented differently between molecules in adjacent unit cells (ab-ba-ab in F35 and ab-ab-ab in F1) and adjacent DNA helices in the lattice are laterally translated (Fig 2A). Nevertheless, like in the P2_1_2_1_2_1_ form, crystal packing interactions remain minimal as contacts between neighbors occur only over the N-terminal β-hairpin arms of Fis and the DNA ends. Fis binds F35 with 75-fold poorer affinity than to F1, whereas binding to the sequence F36, which contains only the +1 and +3 T to C substitutions in the core, is reduced 15-fold (Fig 2B), suggesting that the presence of the ±8(A/T) base pairs lowers Fis binding 5-fold in the context of the F36 binding site.

As expected, the Fis protein structure is essentially identical in the F35 and F1 complexes as the root mean square deviation (RMSD) of all protein atoms is 0.27 Å, and all of the protein-DNA contacts are conserved. However, the DNA structure in F35 differs in several respects. F35 exhibits an asymmetric minor groove width profile over the center of the binding site where the minor groove at the -(2–3) base step is 0.6 Å narrower than at +(2–3) (Fig 2C). The asymmetry reflects the presence of the two C/G base pairs at +1 and +3 that promote wider minor groove widths due to the presence of the guanine 2-amino group [30,34]. This is supported by a lower resolution structure of Fis bound to the F36 sequence that also crystallized in the C2 space group and shows a similar asymmetric minor groove profile (Fig 2C). The Fis-F35 structure also shows differences in its roll angle profile relative to F1. In both structures there is a transition from slightly (–) roll in the center of the binding site (-2 to +2) to positive roll at ±(3/4). Whereas there are distinct (+) roll angle peaks in the F1 structure within the major groove interface, the F35 structure exhibits relatively constant positive roll angles extending from–(7–8) to–(3–4) and from +(3–4) to +(6–7).

The overall DNA curvature in the F35 crystal structure is 66°, the same as in the F1 structure (both measured over the segments from -12 to +12). Also like F1, a small writhe of about (-) 30° is introduced into the DNA. As shown in Fig 2E, the (-) writhe in the DNA follows the basic surface that extends from the core DNA binding interface through both sides of Fis. The correspondence with the electrostatic surface potential of Fis and the similar out-of-plane curvatures in the two crystal forms suggests that the local DNA writhe is not an artifact of lattice packing.

### The major groove interface: role of Y-R steps in Fis binding and DNA conformation

**Fig 3:**
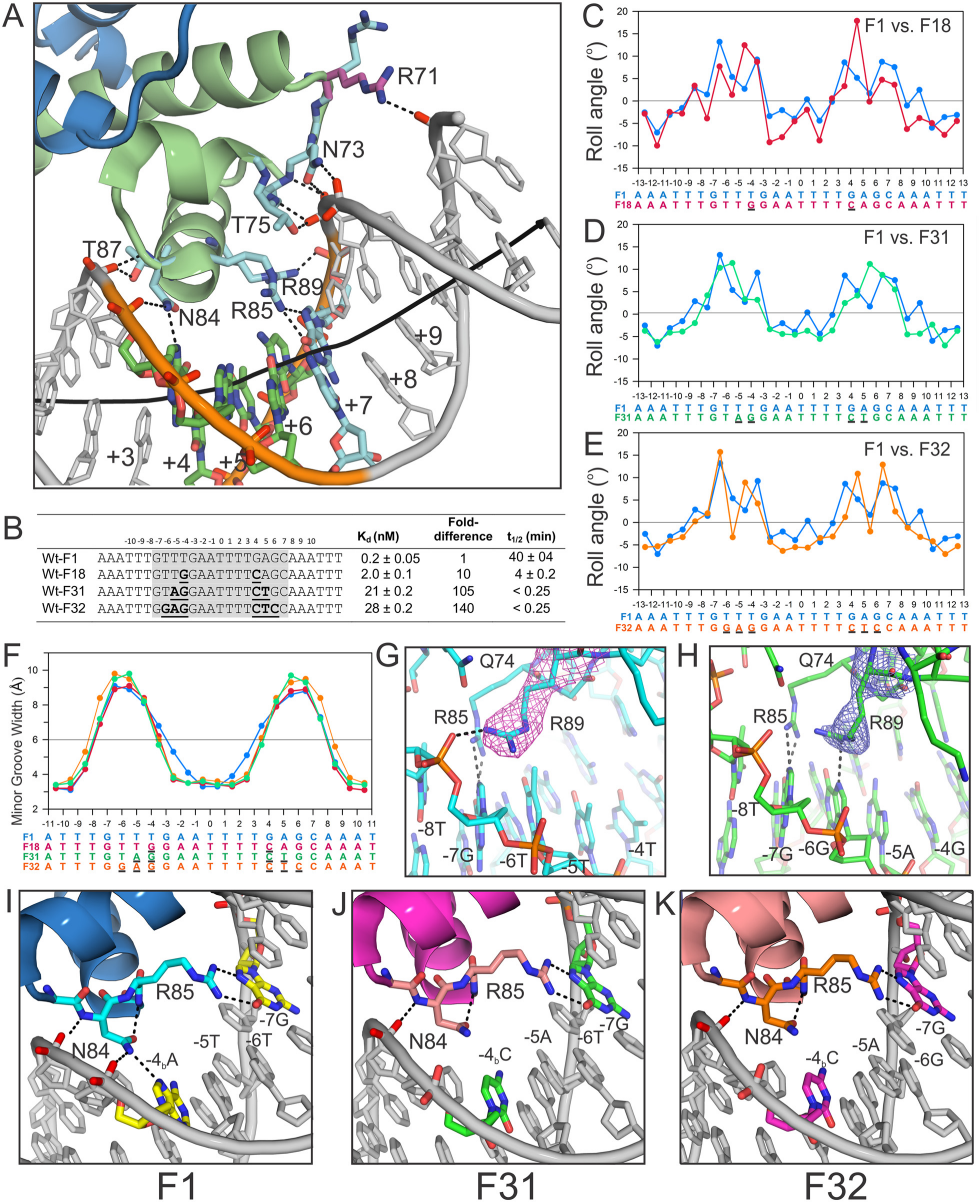
Effects of the flexible ±(3–4) Y-R step on Fis binding, stability, and DNA geometry. **(A**) Zoomed in view of the contacts made to the phosphate backbone in the crystal structure of the reference high affinity Fis-F1 complex. Protein subunits (blue and green cartoon) and DNA chains (grey, except for base pairs 4–6 which are colored by atom) are shown and labeled. The Arg71 side chain has been modeled to contact the phosphate backbone (purple). The structure of Fis-F1, which contains a TG step at +(3–4), is nearly identical to that of Fis-F2 containing a TA step at +(3–4) [28]. **(B)** Sequences, equilibrium binding affinities, and lifetimes for DNA sites with displaced (F18 and F31) or removed (F32) ±(3–4) Y-R steps. Bases that differ from those in the F1 sequence are bold and underlined. **(C-E)** Roll angle plots for each of the major groove Y-R variants relative to F1. Bases that differ from those in the F1 sequence are underlined. **(F)** Minor groove plot for each of the major groove Y-R variants relative to F1. Bases that differ from those in the F1 sequence are underlined. **(G & H)** Position of the Arg89 side chain in the F1 (G) and F32 (H) structures. The 2F_o_-F_c_ electron density map for the Arg89 side chain is shown for the F1 (pink) and F32 (blue) structures at 1.0σ. Dotted lines represent contacts that are within 3.4 Å. **(I-K)** Asn84 contacts in the F1, F31, and F32 crystal structures, respectively. Relevant protein and DNA elements are labeled.

As noted above, high affinity regulatory Fis binding sites are strongly enriched for Y-R steps at the ±(3–4) position [19,21,22]. Of the ~20% of well characterized sites that do not contain a ±(3–4) Y-R step in one or both half-sites, >80% contain a Y-R step shifted to ±(4–5), a feature first noticed by Lazarus and Travers [35]. However, consensus motifs derived from large compilations of reported Fis binding sites and genome-wide binding data do not reflect an overrepresentation of Y-R steps [1,2,20,23]. X-ray structures of Fis complexes with high affinity binding sites containing Y-R steps at ±(3–4) show that the DNA helix axis exhibits (+) roll angle deviations (9–12° for F1, Fig 3D) and low helical twist angles at these positions, consistent with the reduced energetic cost of unstacking Y-R dinucleotide steps [24–27,36]. The bases at ±(3–4) are not directly contacted by Fis with the exception of an H-bond by the Asn84 side chain to ±4 when a purine N7 is present on the bottom strand (Fig 3I–3K). However, loss of this base contact, together with the ±4 backbone phosphate contact, by substitutions of Asn84 to smaller side chains results in little change in equilibrium binding affinities to most high affinity binding sites [22,28,37,38].

To test the importance of the presence and position of a Y-R step for Fis binding and the resulting DNA structure adaptation, we compared the binding properties and X-ray structures of complexes formed on DNA sequences derived from F1 but either lacking a Y-R step (F32) or containing a Y-R step at ±(4–5) (F18, see [28]) or at ±(5–6) (F31) (Fig 3B). F18 exhibits a 10-fold reduction in binding affinity while binding is reduced 100- and 140-fold for F31 and F32, respectively. Moreover, the Fis-DNA complex lifetimes were either too fast to detect by the methods used here (< 0.25 s for F31 and F32) or shortened by 10-fold (4 min for F18) (Fig 3B).

The F31 and F32 complexes crystallized in the same orthorhombic space group as F1 and F18 and were refined at 2.7 and 2.8 Å resolution, respectively (Table 1). The DNA structures exhibit significant differences over the Fis interface, both with respect to the locations of roll angle deviations and minor groove widths. The minor grooves over the centers of the F18, F31, and F32 complexes are narrowed over a more extended region than in the F1 structure (Fig 3F). In each case, the closest phosphates across the minor groove are separated by < 3.6 Å between -3 and +3, whereas F1 and other high affinity complexes containing a ±(3–4) Y-R step exhibit a more gradual narrowing to 3.3 Å at the center of the binding site (Fig 3F). As a consequence of increased DNA minor groove compression and the resulting shift of the backbone at the ±(4–5) step, the Asn84 side chain contacts to the DNA backbone are lost in the F18, F31, and F32 structures (Fig 3I–3K, F18 not shown). Minor groove widths expand rapidly in F18, F31, and F32 between ±(3–6), increasing by up to 6.0 Å. The widened minor grooves correlate with the regions of (+) roll angle changes that are induced by Fis binding within the corresponding major grooves (Fig 3C–3E). Minor groove widths are also increased in the flanking DNA of the Fis-F32 structure, specifically at the ±(7–8) and ±(8–9) phosphates (Fig 3F). As a result, the DNA backbone shifts towards the Fis protein surface, which reorients the Arg89 side chain from the ±(8–9) phosphate to inside the major groove where it is positioned 3.4 Å from the -6G:N7 (Fig 3G and 3H).

**Table 1:** Data collection and refinement statistics.

Structure	Fis-F35	Fis-F1±8A	Fis-F1±8C	Fis-F1±8G	Fis-F31	Fis-F32
PDB code	5E3M	5DTD	5DS9	5E3L	5E3N	5E3O
Data Collection						
Space group	C2	P2_1_2_1_2_1_	P2_1_2_1_2_1_	P2_1_2_1_2_1_	P2_1_2_1_2_1_	P2_1_2_1_2_1_
Beam line	ALS 8.2.1	APS 24-ID-C	APS 24-ID-C	APS 24-ID-C	APS 24-ID-C	APS 24-ID-C
Unit cell dimensions (Å)	*a =* 161.88	*a =* 43.29	*a =* 43.28	*a =* 43.35	*a =* 43.19	*a =* 43.19
	*b =* 43.22	*b =* 93.95	*b =* 93.20	*b =* 93.37	*b =* 92.98	*b =* 94.32
	*c =* 97.16	*c =* 154.50	*c =* 154.28	*c =* 154.68	*c =* 153.95	*c =* 154.34
Unit cell angles (°)	90.0–111.5–90.0	α = β = γ = 90	α = β = γ = 90	α = β = γ = 90	α = β = γ = 90	α = β = γ = 90
Resolution range (Å)	40.4–2.88	80.3–2.56	79.8–2.64	154.6–2.66	93.0–2.66	80.5–2.78
	(3.11–2.88)	(2.70–2.56)	(2.78–2.64)	(2.80–2.66)	(2.80–2.66)	(2.93–2.78)
Completeness (%)	94.4 (99.0)	91.6 (63.5)	96.5 (89.9)	93.7 (97.2)	99.9 (99.9)	92.9 (99.5)
Redundancy	6.6 (7.1)	5.8 (3.7)	3.9 (4.0)	4.8 (5.0)	7.6 (7.3)	5.1 (5.3)
CC_1/2_ (%)	99.8 (80.1)	99.8 (78.5)	99.9 (81.3)	99.7(90.3)	99.6 (91.8)	99.8 (86.2)
R_merge_ (%)	7.9 (85.7)	6.1 (60.4)	4.2 (48.0)	7.6 (60.2)	9.3 (57.8)	6.4 (63.8)
I/σI	13.7 (2.4)	14.7 (2.2)	17.2 (2.7)	11.3 (2.4)	10.3 (2.5)	13.8 (2.6)
Refinement						
Resolution (Å)	2.88	2.56	2.64	2.66	2.66	2.78
No. reflections	13576	19264	18333	17504	18531	15611
R_work_	17.9	22.3	21.6	21.0	21.7	22.0
R_free_	22.6	25.14	25.2	24.9	25.2	26.7
RMSD bond length (Å)	0.02	0.017	0.008	0.005	0.014	0.005
RMSD bond angles (°)	1.78	1.04	1.10	0.954	1.13	0.804
Number of atoms						
Protein	1502	1505	1505	1505	1505	1505
DNA	1101	1101	1101	1101	1101	1101
Water	7	4	5	4	9	10
B factors						
Protein	30.9	45.8	32.3	44.9	31.5	35.5
DNA	60.8	73.1	52.8	68.1	48.7	55.8
Water	15.4	32.2	22.8	27.0	17.8	23.4
Ramachandran statistics						
Favored (%)	98.4	97.8	97.3	98.4	97.3	98.4
Allowed (%)	1.6	2.2	2.7	1.6	2.7	1.6
Generously allowed (%)	0	0	0	0	0	0

Values in parentheses refer to the highest resolution shell.

The positive roll angle peaks at ±(3–4) are displaced in all of the complexes in which the ±(3–4) Y-R step has been altered (Fig 3C–3E). For the F18 and F31 complexes, the roll angle peaks are coincident with position of the displaced Y-R step at ±(4–5) and ±(5–6), respectively (Fig 3C and 3D). The F32 complex containing no Y-R step exhibits a prominent roll angle peak at the ±(5A/T-4G/C) steps as well as the normal ±(6–7) steps. This suggests that (+) roll is required within the major groove interface even at the cost required to unstack a more stable base step (A/T-G/C). Despite the individual differences in local bending across the major groove interfaces, the overall curvatures of the DNAs in each of the crystal structures are similar (65–75°). We also show below that displacement of the ±(3–4) Y-R step does not significantly impact global Fis-induced DNA bending in solution (see below).

### Effects of sequences flanking the 15 bp core recognition motif on Fis-DNA binding and DNA geometry

Sequence compilations, including motif finder analyses of genome-wide binding data, have implicated a preference for A/T-rich sequences flanking the 15 bp core with a mild preference for a T/A bp at ±8 (Fig 1A) [1,2,22]. Moreover, previous studies have suggested that the flanking sequences can affect Fis-induced DNA curvature and binding kinetics [22,29], consistent with the DNA backbone contacts that extend out to ±(9–10) in available crystal structures (Figs 1B and 3A) and probably farther given the role of the R71 side chain in Fis-DNA bending and stability. These observations and the effect of the ±8A substitution on the F35 complex relative to F36 (Fig 2B), motivated us to more systematically investigate the role of flanking sequences on Fis binding, bending, and complex stability.

We first tested the effects of individually substituting base pairs flanking the core at positions ±8, ±9, and ±10 in the context of the high affinity F1 site (Table 2). These data indicate that the base at ±8 can have a large effect on Fis binding with a G being strongly inhibitory (150-fold poorer binding) and an A being moderately inhibitory (10-fold poorer binding). Changes at ±9 or ±10 or further outside the core (e.g., F14, F15, F16) displayed no significant effect on equilibrium binding, and where tested, on lifetimes when a T is present at ±8.

**Table 2:** Effects of flanking DNA substitutions on Fis-DNA binding affinity and complex stability^1^.

	-10 -9 -8 -7 -6 -5 -4 -3 -2 -1 0 1 2 3 4 5 6 7 8 9 10	K_d_ (nM)	Fold-difference^2^	t_1/2_ (min)
F1(±8T)	aaatttGTTTGAATTTTGAGCaaattt	0.2 ± 0.07	1	41 ± 4
F1±8A	aaatt**a**GTTTGAATTTTGAGC**t**aattt	2.1 ± 0.2	10	5 ± 0.5
F1±8C	aaatt**c**GTTTGAATTTTGAGC**g**aattt	0.7 ± 0.2	3	14 ± 1
F1±8G	aaatt**g**GTTTGAATTTTGAGC**c**aattt	30 ± 6	150	< 0.25
F1±9A	aaat**a**tGTTTGAATTTTGAGCa**t**attt	0.5 ± 0.2	3	22 ± 2
F1±9C	aaat**c**tGTTTGAATTTTGAGCa**g**attt	0.3 ± 0.1	2	30 ± 2
F1±9G	aaat**g**tGTTTGAATTTTGAGCa**c**attt	0.6 ± 0.2	3	25 ± 2
F1±10A	aaa**a**ttGTTTGAATTTTGAGCaa**t**ttt	0.5 ± 0.1	3	36 ± 5
F1±10C	aaa**c**ttGTTTGAATTTTGAGCaa**g**ttt	0.3 ± 0.1	2	44 ± 1
F1±10G	aaa**g**ttGTTTGAATTTTGAGCaa**c**ttt	0.5 ± 0.1	3	38 ± 5
F14	**ggg**tttGTTTGAATTTTGAGCaaa**ccc**	0.5 ± 0.1	3	ND^3^
F15	**ccc**tttGTTTGAATTTTGAGCaaa**ggg**	0.4 ± 0.2	2	ND
F16	**gcgg**ttGTTTGAATTTTGAGCaa**ccgc**	0.5 ± 0.2	3	ND
F33	aaa**gg**tGTTTGAATTTTGAGCa**cc**ttt	0.6 ± 0.1	6	ND
F34	aa**gc**ttGTTTGAATTTTGAGCaa**cg**tt	0.2 ± 0.1	1	8 ± 1
INV	**tttaaa**GTTTGAATTTTGAGC**tttaaa**	33 ± 3	165	< 0.25
INV±8G	**tttaag**GTTTGAATTTTGAGC**cttaaa**	250 ± 20	1250	ND
INV±8C	**tttaac**GTTTGAATTTTGAGC**gttaaa**	6.0 ± 2	30	ND
INV±8T	**tttaa**tGTTTGAATTTTGAGCa**ttaaa**	2.3 ± 0.7	12	4 ± 0.7
INV-CAT	**tttca**tGTTTGAATTTTGAGCa**tgaaa**	2.9 ± 1.0	15	ND
INV-GAT	**tttga**tGTTTGAATTTTGAGCa**tcaaa**	2.0 ± 0.6	10	ND
INV±9-10T	**ttta**ttGTTTGAATTTTGAGCaa**taaa**	0.4 ± 0.1	2	ND

^1^Upper case letters represent the 15 bp core Fis binding site sequence and those in lower case represent flanking DNA. Underlined and bold nucleotides highlight those that differ from F1.

^2^Fold-difference relative to the apparent equilibrium dissociation constant (K_d_) for WT Fis with F1 DNA.

^3^Not determined.

Inverting the AT-rich flanking sequence from AAATTT to TTTAAA (INV, Table 2) has a surprisingly large effect on Fis binding as equilibrium binding was 165-fold poorer than F1 and >15-fold poorer than the F1±8A variant. The large effect cannot be explained by disruption of the A-tracts due to introduction of the TA dinucleotide because G/C substitutions within the flanking sequences do not significantly disrupt equilibrium binding (Table 2). When the INV flanking sequence is coupled with a G at ±8 (INV±8G; TTTAAG), binding becomes extremely poor (1250-fold poorer than F1). Taken together, the data in Table 2 suggests that the identity of the dinucleotide at ±(8–9) can significantly modulate Fis binding affinity with the base pair at ±8 having the greatest effect.

**Fig 4:**
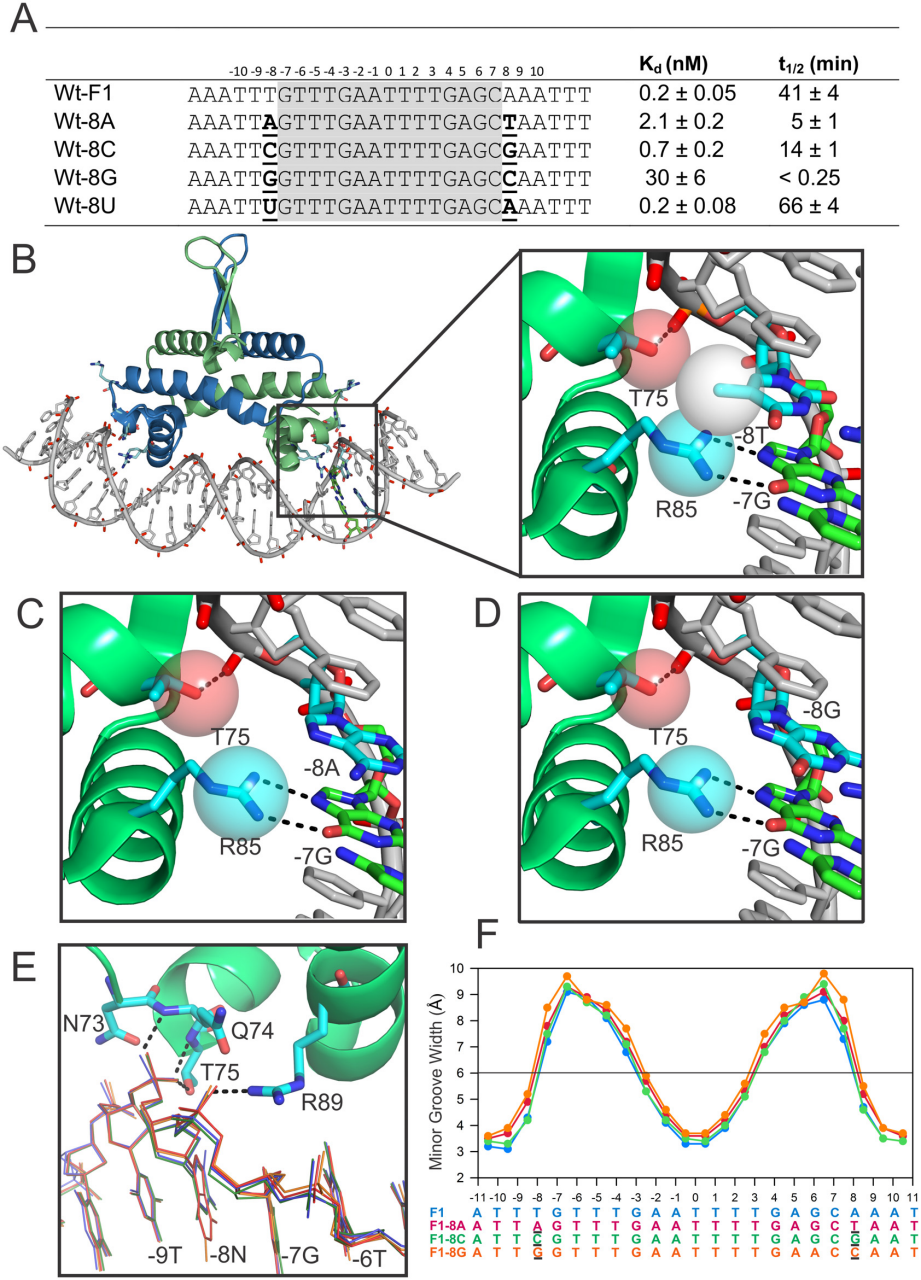
Effects of ±8 substitutions on Fis-DNA binding, stability, and structure. **(A**) Equilibrium binding affinities and lifetimes for DNA sites with symmetric ±8 substitutions. **(B)** Fis-F1 crystal structure highlighting contacts between nucleotides -7G and -8T and Fis residues Thr75 and Arg85. Note van der Waals surface contacts between the -8T C5 methyl and Thr75:Oγ1 and Arg85:CZ. **(C and D)** Same region as highlighted in panel B for the Fis-F1±A (C) and Fis-F1±G (D) crystal structures. Van der Waals contacts to the -8 base are absent in these as well as the F1±8C structure (not shown). **(E)** DNA backbone positional variations in the Fis-F1 (blue), Fis-8A (red), Fis-8C (green) and Fis-8G (orange) structures. Structures were aligned over the Fis proteins. Only Fis-F1 side chains that contact the flanking DNA backbone are shown. Contacts within 3.4 Å are represented by dotted lines. **(F)** Plot of minor groove widths for each of the ±8 variants. Bases that differ from those in the F1 sequence are underlined.

We determined X-ray structures of Fis complexes for each of the ±8 variants to elucidate the structural origins of the observed binding and lifetime differences. There are few differences in the overall structures of the proteins (RMSDs within 0.27 Å) and DNAs (RMSDs within 0.36 Å) in pairwise comparisons between the F1, F1±8C, F1±8A, and F1±8G complexes. Significantly, the locations of the ±8 phosphates in each of the variants are nearly identical to F1, which is expected since each of the non-esterified oxygens at this position are H-bonded to peptide amides from Gln74 and Thr75 at the N-terminal end of helix C (Figs 1D and 4E). In contrast, the phosphates at positions ±7 and ±6 are shifted by up to 0.6 Å in F1±8G and F1±8A, but by only 0.2 Å in the F1±8C complex (Fig 4E and 4F). We suggest that a G/C or A/T bp at ±8 may increase the cost required to assume a DNA conformation that allows for the formation of the critical contacts to the ±8 phosphate. The magnitude of the shift in the DNA backbone between ±6 and ±8 correlates qualitatively with the effects of the ±8 substitutions on binding affinity, complex stability, and DNA bending (Fig 4A and see below).

Another notable difference between the F1 and each of the ±8 variant structures is the van der Waals contact with the ±8 thymine methyl group made by the Arg85 guanidinium group and the Thr75 γ-hydroxyl group (Fig 4B). These contacts are absent in the F1±8A, F1±8C, and F1±8G structures, but the positions of the Arg85 and Thr75 side chains are unchanged (Fig 4C and 4D). To test the functional importance of the contacts to the ±8 thymine methyl group, we performed equilibrium binding and lifetime experiments for a DNA duplex substituted with uracil at the ±8 position. Removing the ±8 thymine methyl has no measurable effect on equilibrium Fis binding affinity, and surprisingly, the uracil-substitution significantly increases lifetimes of the Fis-DNA complexes (Fig 4A). We conclude that the presence of the methyl group on ±8 thymine does not appear to enhance binding, at least in the context of the optimal F1 core sequence.

### DNA shape features of the core recognition sequence determine importance of flanking Fis-DNA contacts

Structural and biochemical data show that the side chains of Asn73, Thr75, and Arg71 contact to the DNA backbone flanking the 15 bp core (Figs 1B–1D and 3A, Table 3) (see also [22,28,37]). Asn73, which contacts both the ±8 and ±9 phosphates, is particularly important, and as expected, Fis-N73A exhibits defective binding even to the high affinity site F1 (Table 3). On the other hand, alanine substitutions at Arg71 or Thr75 have little effect on equilibrium binding to F1. However, Fis-T75A is unable to bind to F28A, which contains a wider minor groove at the center of the core due to three G/C base pairs [30], and is even compromised for binding to F27 that contains only a single G/C base pair in the central region and is bound with high affinity by Fis-wt. Fis-R71A is strongly compromised for binding to Fis sites F27 and F28 containing G/C base pairs at their center as well as to F1±8G. Fis-T75A or Fis-R71A do not exhibit synergistic effects with YR mutations (Table 3). We conclude that Fis-DNA contacts outside the core recognition region can have a strong effect on binding affinity depending upon the DNA shape features of the target sequence.

**Table 3:** Interplay between Fis residues contacting the flanking sequences and binding site variants.

	-10 -9 -8 -7 -6 -5 -4 -3 -2 -1 0 1 2 3 4 5 6 7 8 9 10	Fis protein	K_d_ (nM)	Fold-difference^1^
**F1**	aaatttGTTTGAATTTTGAGCaaattt	WT	0.2 ± 0.05	1
		R71A	0.5 ± 0.2	2.5
		T75A	0.3 ± 0.8	1.5
		N73A	29 ± 0.2	140
**F27**	aaatttGTTTGAA**C**TTTGAGCaaattt	WT	0.2 ± 0.1	1
		R71A	2.8 ± 0.5	14
		T75A	3.5 ± 0.9	18
**F28**	aaatttGTTTGA**GCG**TTGAGCaaattt	WT	28 ± 4	140
		R71A	470 ± 100	2300
		T75A	> 1000	> 5000
**F32**	aaatttG**GAG**GAATTTT**CTC**Caaattt	WT	28 ± 5	140
		R71A	73 ± 11	370
		T75A	76 ± 10	380
**F1±8G**	aaatt**g**GTTTGAATTTTGAGC**c**aattt	WT	30 ± 0.8	150
		R71A	450 ± 20	2270
		T75A	48 ± 5	240

^1^Fold-difference relative to the apparent equilibrium dissociation constant (K_d_) for WT Fis with F1 DNA.

### DNA curvatures associated with Fis-DNA complexes are controlled by flanking sequences

Flanking sequences have been found to influence the magnitude of Fis-induced DNA bending [22], although the sequence determinants that underlie these effects have not been elucidated. In order to further investigate impacts of flanking sequence on curvatures of free and Fis-bound DNA, we have used polyacrylamide gel mobility and in-gel Förster resonance energy transfer (FRET) to measure Fis-induced bending for DNA sites containing substitutions in the flanking DNA. The gel migration assay, which employs 422 bp fragments, reports on global bending in the Fis-DNA complex, whereas the FRET assay, which employs 27 bp fragments, reports on local bending differences (see below).

#### Global curvatures evaluated by electrophoretic mobility

**Fig 5:**
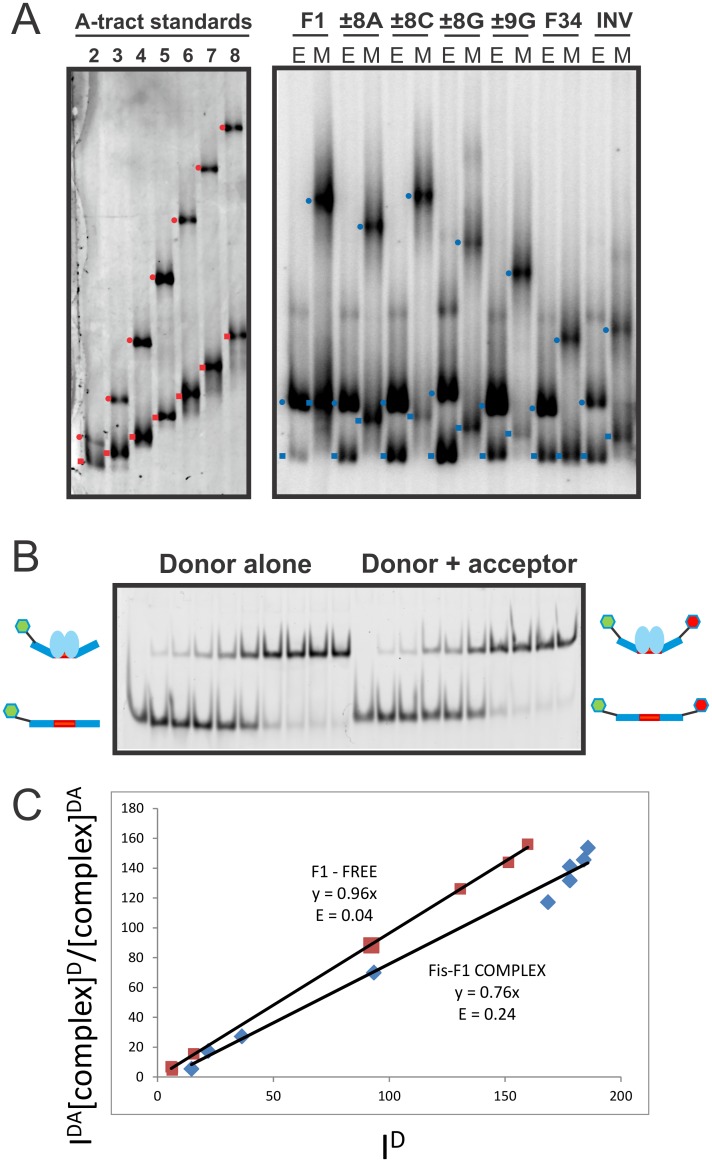
Effects of DNA base substitutions on DNA bending by Fis. **(A**) Electrophoretic mobility shift assays showing migrations of free and Fis-bound DNA fragments. DNA fragments containing two to eight in-phase A-tracts were used as standards for estimating bend angles ([41]; left panel). Faster migrating fragments contain A-tracts at the end of the fragment (red squares), whereas slower migrating fragments contain A-tracts in the middle (red circles). Right panel: Fis-DNA complexes formed on 422 bp DNA fragments in which the Fis binding sites were located near the end (E) or the middle (M) of the fragment. Bands corresponding to unbound fragments (blue squares) and Fis-bound fragments (blue circles) are marked. **(B)** Representative FRET gel for Fis binding to F1 DNA. The intensity of the donor fluorophore emission in the absence (left titration) or presence (right titration) of the acceptor fluorophore (Alexa Fluor 555) was quantified from a scan of the gel where λ_ex_ = 488 nm and fluorescence emission was collected through a 520 nm emission filter. The same gels were scanned by phosphorimaging as described in the Methods (not shown). Cartoons describe the species that correspond to the bands on the gel. The left titration is that for a donor-only labeled DNA (“D”; blue rectangle with the Fis site shown in red). The donor fluorophore (Alexa Fluor 488 –green hexagon) is conjugated to the 3′ end of the DNA oligonucleotide by a 6 carbon linker (black line). The free DNA is shifted to the upper band when bound by the Fis dimer (light blue ovals). The right half of the gel represents the titration of the donor and acceptor labeled DNA (“DA”; Acceptor is Alexa Fluor 555 –red hexagon). **(C)** A plot showing the correlation between the fluorescence intensities of the donor-acceptor labeled DNA (I^DA^) and donor-only labeled F1 DNA (I^D^) in the presence (blue diamonds) and absence (red squares) of Fis. The plots were fit to a line with the equation I^DA^[(complex^D^)/(complex^DA^)] = I^D^(1 –E), where E is FRET efficiency and the slope is (1-E) as described in the Methods. Axis units are x1000.

For the gel migration bending assay, global bend angles of the free and Fis-bound sites were calculated by measuring the relative electrophoretic migrations of 422 bp fragments containing a Fis site in the middle and at the end of the fragment [22,39–41]. The ratio of these migration distances was compared to those of standards containing 2–8 A-tracts in helical phase with each other [41]. The F1 site contains three in-phase A-tracts: AAATTT at each flank and AATTTT at its center. As expected, the F1 site exhibits considerable intrinsic curvature (~50° assuming 18° per A-tract, Fig 5A; Table 4). The apparent DNA curvature increases dramatically upon Fis binding (~120°) indicating that Fis binding induces a substantial amount of additional DNA bending relative to the free DNA (Fig 5A; Table 4). As noted previously [22], and shown here with F34, the presence of an intrinsically bent target site is not required for high affinity Fis binding.

**Table 4:** Effects of flanking and core substitutions on Fis-induced DNA bending.

		Gel mobility assay		In-gel FRET assay			
		(Bend angle °)		(FRET efficiency)			
		Free	Complex	Free	Complex	Distance (Å)	Angle (°)
	-10 -9 -8 -7 -6 -5 -4 -3 -2 -1 0 1 2 3 4 5 6 7 8 9 10					(complex)^1^	(complex)^2^
F1	aaatttGTTTGAATTTTGAGCaaattt	51 ± 3	119 ± 3	0.09 ± 0.01	0.27 ± 0.01	82.6	68
F1±8A	aaatt**a**GTTTGAATTTTGAGC**t**aattt	48 ± 3	109 ± 3	-	-	-	-
F1±8C	aaatt**c**GTTTGAATTTTGAGC**g**aattt	47 ± 3	126 ± 2	0.13 ± 0.01	0.26 ± 0.01	83.3	66
F1±8G	aaatt**g**GTTTGAATTTTGAGC**c**aattt	41 ± 1	99 ± 1	0.09 ± 0.01	0.23 ± 0.01	85.6	61
F1±9A	aaat**a**tGTTTGAATTTTGAGCa**t**attt	46 ± 3	104 ± 2	-	-	-	-
F1±9C	aaat**c**tGTTTGAATTTTGAGCa**g**attt	45 ± 1	99 ± 1	-	-	-	-
F1±9G	aaat**g**tGTTTGAATTTTGAGCa**c**attt	42 ± 2	89 ± 2	0.08 ± 0.03	0.18 ± 0.04	90.1	50
INV	**tttaaa**GTTTGAATTTTGAGC**tttaaa**	≤ 36	62 ± 1	0.06 ± 0.04	0.14 ± 0.01	94.7	36
F34	aa**gc**ttGTTTGAATTTTGAGCaa**cg**tt	~ 0	62 ± 3	0.12 ± 0.02	0.26 ± 0.02	83.3	66
F18	aaatttGTT**G**GAATTTT**C**AGCaaattt	51 ± 2	116 ± 1	-	-	-	-
F31	aaatttGT**AG**GAATTTT**CT**GCaaattt	51 ± 2	108 ± 2	-	-	-	-
F32	aaatttG**GAG**GAATTTT**CTC**Caaattt	52 ± 1	106 ± 2	-	-	-	-

^1^Inter-fluorophore distance calculated from FRET efficiency as detailed in the Methods.

^2^Angle calculated assuming a single central bend in the Fis-bound DNA as detailed in the Methods.

DNA substitutions at the ±8 position exhibit variable effects on free and Fis-induced DNA bending (Fig 5B, Table 4). The ±8A and ±8C substitutions show little effect on intrinsic bending, but the ±8G substitution reduces the apparent intrinsic DNA curvature by about 20%. DNA curvature of the Fis-bound complex is reduced by 12 and 17% for the ±8 purine substitutions (±8A and ±8G, respectively), whereas bending by the ±8C substitution is similar to F1. The greater curvatures of Fis complexes when a pyrimidine is at ±8 may be attributable to the presence of a flexible Y-G dinucleotide at ±(7–8). Substitutions at ±9 have a modest effect on free DNA bending (decreased 8–17%) and a more significant effect on bending in the Fis-DNA complex (decreased 16 to 28%) (Table 4). The ±9 substitutions displace the flanking A-tracts such that the three A-tracts in the F1 site are no longer in perfect helical phase with the central A-tract, explaining the reduction of curvature in the unbound fragment. The larger bending reduction in the complex implies that these substitutions also inhibit association of the flanking DNA with the basic sides of the Fis dimer. The F34 site, which does not contain flanking A-tracts due to the G-C substitution at ±(10–11), eliminates intrinsic bending and decreases Fis-induced bending by half (Fig 5A; Table 4). Finally, the INV binding site, which maintains the same A/T composition but both converts the flanking segments to non-A-tract sequences and removes the ±(7–8) Y-G steps, reduces intrinsic and Fis-induced bending by ≥ 30% and 50%, respectively. Taken together, these data indicate that global Fis-induced curvature is influenced by sequence determinants in the flanking DNA: maximal bending angles are generated when a pyrimidine is at ±8, creating a Y-G step, and when the flanking DNA contains an intrinsically bent A-tract that is in helical phase with the center of the core.

#### Local curvatures evaluated by FRET

Fis-induced bending was also probed by in-gel FRET utilizing 27 bp DNA oligonucleotides labeled with donor fluorophore alone (Alexa Fluor 488), or donor and acceptor (Alexa Fluor 555; Fig 5B). The donor labeled strand was radiolabeled with ^32^P and used for both donor alone (D) and donor-acceptor duplexes (DA); thus each duplex had the same ^32^P specific activity, and the relative concentrations of D and DA complexes could be calculated by phosphorimaging. Corrected donor emission of the isolated Fis-DNA complex and free DNA species was quantified in the presence of acceptor and was plotted against donor emission measured in the absence of acceptor (Fig 5C; see methods). A slope of < 1 is achieved when there is resonance energy transferred from the donor to the acceptor such that:

$${\text{I}}^{\text{DA}}{\text{[(complex}}^{\text{D}}{\text{)/(complex}}^{\text{DA}}{\text{)] = I}}^{\text{D}}\left(\text{1 – E}\right)$$ (1)

where I^DA^ and I^D^ are donor emission intensities in the presence and absence of acceptor, respectively, (complex)^D^/(complex)^DA^ is the ratio of complex D to DA as measured by phosphorimaging, and E is FRET efficiency.

Although the FRET assay only reports the distance between the ends of the 27 bp duplexes used and is therefore limited in its dynamic range, the results generally mirror those obtained by gel migration (Table 4). The FRET efficiency for the Fis-F1 complex is 0.27, which corresponds to an inter-fluorophore distance of 83 Å (assuming an R_0_ of 70 Å for the Alexa Fluor 488/555 pair) and a 68° bend angle based on this end-to-end distance (see Materials and Methods for angle calculation). This angle is strikingly similar to what is observed in the Fis-DNA crystal structures generated with the same length oligonucleotides but is considerably less than the 119° measured by gel migration assays. We believe this difference is because the longer DNA molecules used in the gel assays are able to “wrap” around the basic surface of the Fis protein resulting in further shortening of the DNA end-to-end distance and consequently greater retardation of the electrophoretic migration.

Both the FRET and gel migration assays report no differences in curvature by the ±8C substitution but significant reductions by the ±8G and ±9G substitutions (Table 4). Inverting the flanking sequence (INV) reduced Fis-induced FRET efficiency by about 50%, and the ±9G substitution reduced FRET efficiency by 33%, again in general agreement with the gel migration assays. On the other hand, although disrupting the flanking A-tracts in F34 with the ±(10–11) G-C substitution strongly reduces bending measured by gel migration, values measured by FRET are nearly identical to those observed for F1. We attribute this difference to the fact that these substitutions are too close to the labeled ends to reveal differences by FRET.

#### Effects on bending by Y-R substitutions within the core

As discussed above, X-ray structures revealed that the presence and positions of a Y-R step in the major groove interfaces altered the locations of roll angle deviations at the dinucleotide level, but the local DNA curvatures over the interface were similar to that of F1. We asked whether gel mobility assays would reveal differences in long range curvatures in complexes formed on the Y-R mutants. As shown in Table 4, intrinsic curvatures of the Y-R mutants were unchanged from F1, and the Fis-bound complexes exhibited only small differences relative to F1 (9 and 11% for F31 and F32, respectively). The apparent bending angle by the F18 (Y-R at ±(4–5)) complex was essentially indistinguishable from F1, even though F18, like the other Y-R mutants, exhibited variations in both local bending and minor groove widths. We conclude that the curvature over the core Fis interface is largely constant even though the DNA molecules are adapting differently to the Fis surface at the atomic level as a function of their sequence.

## Discussion

Fis is remarkable in its ability to bind with high affinity to DNA sites that are poorly related at the sequence level. Work described in this report builds on earlier studies to give a more complete understanding of the mechanism of DNA target selection by Fis and the biochemical and structural properties of the resulting complexes. In particular, we have gained new insights into the roles of Y-R steps within the major groove interface and the roles of sequences outside of the generally considered 15 bp core binding motif.

Our current understanding of how Fis selects high affinity targets is as follows: Fis scans the genome through non-specific electrostatic forces until it encounters a DNA segment with an intrinsically narrow or dynamically compressible minor groove over about a half helical turn. A narrow minor groove is required to accommodate the closely spaced recognition helices that are separated in the Fis dimer by a distance that is 8–10 Å shorter than the pitch of canonical B-DNA [42,43], thereby enabling Fis to insert into adjacent major grooves [28]. In all 21 structures of Fis-DNA complexes solved to date, the minor groove is compressed to about half the width of normal B-DNA at the center of the binding site; the key molecular determinant for this compression is the absence of guanine 2-amino groups, which protrude into the minor groove (this paper, [28,30]). The F35 structure described here shows that the central minor groove within a Fis complex can be asymmetrically compressed because of asymmetrically positioned G/C base pairs. The minor groove is not directly contacted by Fis and the central 5 bp segment remains relatively straight.

We propose that the next critical step towards forming a Fis-DNA complex is the bending of DNA within the major groove interfaces in order to establish critical contacts to DNA backbone phosphates between ±7 and ±10, and for high affinity sites, a guanine at ±7. The most extensive phosphate interactions occur at the 5′ phosphate of the ±8 nucleotide where peptide amides from two residues (Gln74 and Thr75) and side chain groups from two residues (Asn73 and Thr75) hydrogen bond to the non-esterified oxygens (Fig 1D). The essential nature of these contacts to the ±8 phosphate are supported by previous ethylation interference assays [44]. Fis-induced DNA bending required to generate these contacts shortens the distance between the ±8T P atoms to 45.4 Å in the Fis-DNA complex from 58.0 Å in canonical B-DNA and is driven by modest (+) rolls at variable positions within the major groove interfaces that depend on the particular sequence. The DNA conformation that generates the most stable complexes contains (+) roll peaks at ±(3–4) and ±(6–7). The peak at ±(3–4) is located at a flexible Y-R step that is prone to (+) rolls [24,26]. The ±(6–7) peak is stabilized by Arg85, which H-bonds to the conserved guanine at -7t and +7b and sometimes also to the ±6 base pair. The ±7G is the only highly conserved base within high affinity Fis sites and engages in the only critical base contact in the complex.

DNA binding sites that do not contain a Y-R dinucleotide at ±(3–4) adapt by different structural mechanisms to form lower affinity and less stable complexes. For example, the complete absence of Y-R steps within the major groove interfaces (F32) results in extremely unstable complexes with a 30-fold poorer equilibrium binding constant. The DNA in the complex maintains the ±(6–7) roll peak, but the second kink is shifted to ±(4–5). A Fis-DNA complex formed on a sequence containing Y-R steps at ±(5–6) (F31) contains a single broad peak of (+) roll that spans 2 base steps at ±(5–7). This complex also exhibited short lifetimes with a 20-fold poorer equilibrium binding constant. In addition, a complex formed on a DNA containing Y-R steps at ±(4–5) (F18) formed less stable complexes with a 10-fold poorer equilibrium binding constant and with prominent (+) roll peaks at ±(4–5). Molecular bending within the major groove interfaces can be asymmetric as demonstrated by the F35 complex where regions of (+) roll angle deflections range from the–(3–4) to–(7–8) in one half-site and from +(3–4) to +(6–7) in the other. Similarly, in the F1 complex, the peak roll in one half-site is localized to -7G-6T, but extends from +6G to +8T in the other. These structures illustrate how different DNA sequences adapt to the Fis surface at the atomic level. Importantly, however, even though the positions and magnitudes of the bends are variable, the overall curvatures of the DNAs over the primary Fis interface are all similar as determined by crystallography and electrophoretic mobility.

A second important feature of a Y-R step at ±(3–4) is its effect on minor groove widths. The minor groove width rapidly switches from compressed to expanded at the ±(3–4) Y-R step in high affinity complexes. However, when the Y-R step is shifted outward or absent entirely, the minor groove remains narrow for an additional base pair on either side (Fig 3F). The ±(3–4) Y-R steps in the high affinity F1 (both TG) and F2 (TA and TG) complexes adopt low helical twist angles (27–30°), positive rolls (7°-10°), and positive slide (0.3–0.6 Å), which all contribute to widening of the minor groove [28]. When the ±(3–4) Y-R dinucleotide is shifted outward (F18 and F31) or absent (F32), these structural features also shift together with the change in minor groove widths.

We show that the identity of the ±8 bp also has a significant effect on Fis-DNA complex affinity, stability, and bending, which correlates with the multiple contacts with the ±8 phosphate (Fig 1D, Tables 2 and 3). Again, a Y-R step, where the R is the conserved ±7G, is optimal as F1 derivatives with TG or CG at ±(7–8) exhibit the highest binding affinities, longest lifetimes, and greatest overall DNA curvatures. A guanine at ±8 is particularly detrimental for Fis binding as complexes are very unstable and bending is reduced even in the context of the otherwise optimal F1 sequence. The DNA within the crystal structures of F1±8G as well as F1±8A reveal up to a 0.6 Å shift in the DNA backbone at the ±(6–7) and ±(7–8) phosphates, the latter of which is contacted by Fis-Arg89. The functional importance of the ±8 position relative to other flanking positions argues that it should be considered as part of the Fis binding motif as shown in Fig 1A.

Although the DNA over the core interface is rigidly bound to Fis, the segments extending out from ±9 are dynamically associated with the track of positive electrostatic potential on the sides of the protein to give variable overall curvatures depending on the intrinsic structural features of the flanking sequences (Fig 2E) (this paper, [22]). Global DNA curvatures are particularly large when the flanking segments contain A-tracts extending out from ±8 or ±9, which would place them in helical phase with the central A/T-rich segment (Table 4). The Fis-F1 complex containing A-tracts in both flanks is bent by ~120°, as estimated from the gel-based bending assays. Complexes formed on other Fis binding sites have been reported to range from 95° down to 45° [22,33,40,41]. Angle determinations based on gel mobilities may be subject to artifacts [45], but we note that measurements of complexes to the F1 sequence without flanking A-tracts (e.g., INV and F32, both at 62°) are close to the 66° observed in crystals and the 68° estimation from FRET measurements on 27 bp F1 duplexes.

Flanking A-tracts may contribute to increased DNA bending and complex lifetimes in two distinct and cooperative ways. First, A-tracts are intrinsically curved toward the minor groove [46], i.e., toward the Fis surface, which facilitates electrostatic interactions between Fis and DNA. Second, the narrow minor grooves of A-tracts [47], as observed in the flanking DNA in the Fis structures (Figs 2C and 3C), exhibit increased electronegativity [48], thereby further enhancing electrostatic interactions with Fis. DNA wrapping around the basic sides of Fis stabilizes the Fis-DNA complex, as evidenced by the fast off-rates of Fis-R71 mutants [22,33] and can compensate for weak core interactions (Table 4). We note that, whereas A/T composition is the important sequence characteristic in the central minor groove segment, a bona fide sequence fitting the definition of an A-tract with no T-A steps appears to be optimal for the flanks. Thus, TTTAAA in place of AAATTT in both flanks results in a 30-fold poorer equilibrium binding constant (Table 2) and low overall curvature (Table 4), whereas similar substitutions within the center of the core have little effect on binding affinity or DNA structure in the Fis complex [28]. Significantly, in a compilation of regulatory Fis sites about half contain an A-tract within the flank of one or both half-sites. The presence of an A-tract upstream of the native H′ IHF binding site has also been found to increase IHF-DNA complex affinity and bending [49–51]. It has also been suggested that this enhancement is driven by recognition of the intrinsic structural features of the A-tract, most importantly its narrow minor groove [49].

Fis-Arg71 is a particularly important Fis residue controlling global curvatures as substitution of Arg71 with any residue except lysine strongly reduces global Fis-induced DNA curvatures and lifetimes [22,33]. In the case of the F1 site we estimate a 12% reduction in global curvature by Fis-R71A by gel mobility assays, but larger effects on global curvatures are observed at sites without flanking A-tracts [22,33]. Arg71 contacts the ±13 phosphate (Fig 1B and 1C), which is supported by site-directed DNA scission by chemical nucleases tethered to residue 71 [22]. There is a complex combinatorial relationship on binding affinity between contacts within the flanking sequence, including those contacted by Arg71, Asn73, and Thr75, and the DNA sequence within the core. When the core sequence is optimal, as in the F1 site, loss of the Arg71 and Thr75 flanking contacts by alanine substitutions have little effect, but with G/C substitutions in the center binding is strongly inhibited or virtually abolished (Table 3). The importance of the Asn73 side chain, which contacts both the ±8 and ±9 phosphates, is highlighted by the fact that the N73A substitution results in a 140-fold reduction in binding affinity in the context of the optimal F1 site. Similar observations have been made for Fis binding to native sites where Asn73, Thr75 and Arg89 mutants bind with variable affinities depending on the binding site [37,52].

In summary, Fis selects its high affinity targets primarily through indirect mechanisms involving a series of positive and negative determinants affecting DNA structure (Fig 1A). Positive structural determinants include: 1. A/T base pairs within the center of the binding site, which allows for the required minor groove compression, 2. a Y-R step at ±(3–4), which facilitates optimal conformation to the Fis surface within the major groove interface to enable contacts to the backbone of the flanking DNA and to guanines at ±7, 3. a Y-G step at ±(7–8) to facilitate DNA backbone interactions at the edge of the primary interface, and 4. A-tracts within the flanking sequence and a phosphate contact with Arg71 to facilitate wrapping of the DNA along the basic sides of Fis. Negative structural determinants include: 1. G/C base pairs within the center of the binding site that inhibit minor groove compression, 2. a C or T at ±3, which probably inhibits formation of the optimal DNA structure (see [28,29]), 3. adenines at ±4 because the thymine methyl on the complementary strand clashes with the Asn84 side chain [28,29], and 4. purines, especially guanines, at ±8, which inhibit formation of critical backbone contacts.

The DNA shape conformed by Fis is important for its biological activities. For example, the serpentine shape of the Fis-bound Hin enhancer is required for invertasome assembly during site-specific DNA inversion [53]. Fis recruits phage λ Xis, a winged-helix DNA binding protein, to an overlapping site within the *attR* locus to promote viral excision from the chromosome [32,54]. Recent co-crystal structures of Fis+Xis bound to DNA indicate that Fis-induced changes in minor groove widths are the key determinant mediating Fis-Xis binding cooperativity (S.P.H. and R.C.J., unpublished), and the resulting change in DNA trajectory is critical for excisive intasome assembly [55]. Fis activates transcription at promoters that contain high affinity Fis binding sites by recruiting the RNA polymerase alpha subunit C-terminal domain (αCTD) [33,56]. The αCTD binds within the flanking DNA segment that associates with the sides of Fis, and Arg71 switches from contacting flanking DNA to interacting with the αCTD [11,12]. Modeling based on an αCTD-DNA structure [57] indicates that the DNA flanking the Fis core would need to be less curved with a wider minor groove than present in the Fis-DNA crystals. Fis can also indirectly regulate transcription through changes in DNA architecture that enhance or inhibit interactions between regulators and RNA polymerase [5]. Finally, we note that Fis-mediated bending leading to compaction of long DNA molecules, as observed by single-DNA molecule studies, is proposed to contribute to chromosome packaging within the bacterial nucleoid [18].

## Materials and Methods

### Crystallization and Structure Determination

Fis and DNA were co-crystallized as described previously [28]. DNA for crystallography was obtained from IDT. Data for all structures except F35 and F36 were collected at 100 K at the Advanced Photon Source (Chicago IL) beamline 24-ID-C (Table 1); F35 and F36 were collected at the Advanced Light Source, Berkeley CA, beamline 8.2.1. Data were processed using AUTOPROC [58] with XDS [59] for integration and AIMLESS [60] for scaling. Anisotropy scaling was performed using the NIH Diffraction Anisotropy Server (http://www.doe-mbi.ucla.edu/sawaya/anisoscale) [61]. Fis-DNA structures were solved by molecular replacement (PHASER) [62] using the Fis-F1 structure (PDB ID: 3IV5) as the search model (see [28]). The models were refined using PHENIX [63,64] and BUSTER [65] and additional model building and validation was done in COOT [66]. Data collection, refinement statistics, and PDB codes for the new structures are given in Table 1. A data set of the Fis-F36 complex was partially refined at 3.5 Å and used to compare minor groove widths with F35 (Fig 2C). DNA structure analyses were performed using the 3DNA suite [67] and DNA curvature was measured with CURVES [68]. Electrostatic potential calculations were performed using the AMBER force-field as implemented by the PDB2PQR server [69], and the Poisson-Boltzmann equation was solved using APBS [70]. Structure figures were generated using PyMol (http://www.pymol.org). Because direct comparisons of the new structures were made with that of Fis-F1, the Fis-F1 structure was re-refined in the same manner as the query structures. This refinement protocol yielded a Fis-F1 model that was indistinguishable from the 3IV5 structure deposited in the PDB.

### DNA Binding and Lifetime Assays

Equilibrium binding was measured by electrophoretic gel mobility shift assays (EMSA) in the following binding buffer: 0.15 M NaCl, 20 mM Hepes (pH 7.5), 5% glycerol, 0.5 mg/ml BSA, 1 mM EDTA, 1 mM DTT, 0.1 nM ^32^P-27 bp binding site probe and 50 μg/ml poly dI/dC. Lifetime (T_1/2_) measurements were performed by adding ≥10,000-fold molar excess of unlabeled F1 duplex to preformed ^32^P-DNA—Fis complexes in the above buffer and following the time-dependent decay of the labeled complex by EMSA [22,28]. Fis-DNA complex was plotted on a semi-log scale as a function time and fit to a linear regression where T_1/2_ was is the time required for half of the complex to decay. Fis protein was prepared as described previously [28]. DNA for binding assays was obtained from IDT.

### DNA bending measurements by polyacrylamide gel migration

Bending angles were measured by gel migration as described [41]. Briefly, Fis-DNA complexes were formed on 422 bp restriction fragments from pCY4-derivatives [71] containing the respective 27 bp Fis site cloned between the SacI and BglII restriction sites. EcoR1 digestion places the Fis binding site near the end and EcoRV digestion places the Fis site near the center of the fragment. Fragments were treated with antarctic phosphatase and 5′ radiolabeled with polynucleotide kinase (New England Biolabs) and γ-^32^P-ATP. Complexes were formed in binding buffer (above) in the presence of 50 μg/ml poly dI/dC competitor DNA. Native PAGE was performed at 5 V/cm on a 6% 29:1 acrylamide:bis-acrylamide gel. The relative mobility of the complexes containing the Fis binding site at the end or middle of the fragment (μm/μe) was compared to that measured for fragments containing two to eight in phase A-tracts positioned at either the end, or middle, of a DNA fragment [41]. Bend angles in the Fis-DNA complex were estimated from the μm/μe ratio assuming that each A-tract contributed 18° of helical bending [47,72].

### DNA bending by FRET

In-gel FRET assays were carried out essentially as described by Radman-Livaja et al. [73]. Briefly, to prepare the DNA duplexes, succinimidyl esters of Alexa Fluor 488 (donor) and Alexa Fluor 555 (acceptor) (Life Technologies) were post-synthetically conjugated to an amine-modified 6-carbon linker appended to the 3’ end of the top and bottom strands (IDT), respectively as per the supplier recommendations. Each strand was PAGE purified and the top strand was 5′-^32^P-radiolabeled and annealed to an unlabeled (D) or acceptor-labeled (DA) bottom strand. DNA duplexes were incubated with increasing amounts of Fis protein in binding buffer (above), and Fis-DNA complexes were separated from free DNA by native PAGE. Donor emission was quantified for free and Fis-bound complexes in the presence (I^DA^) and absence (I^D^) of acceptor on a Typhoon imager by exciting the gels at 488 nm and measuring emission through a 520 nm filter. FRET efficiency (E) was calculated from a plot of the ^32^P corrected donor emission against donor emission measured in the absence of acceptor as follows:

$${\text{I}}^{\text{DA}}{\text{[(complex}}^{\text{D}}{\text{)/(complex}}^{\text{DA}}{\text{)] = I}}^{\text{D}}\left(\text{1 – E}\right)$$ (1)

where I^DA^ and I^D^ are donor emission in the presence and absence of acceptor, respectively, (complex)^D^/(complex)^DA^ is the ratio of complex D to DA as measured by phosphorimaging, and E is FRET efficiency. A slope of < 1 is achieved when there is resonance energy transferred from the donor to the acceptor. It should be noted that no acceptor labeling correction was applied as acceptor labeling was measured to be > 98% efficient. Inter-fluorophore distances were calculated from FRET efficiency (E) as follows,

$$E=\frac{R_{0}^{6}}{R_{0}^{6}+r^{6}}$$ (2)

where r is the distance separating, and R_0_ is the Förster distance for the donor acceptor pair (70 Å; Life Technologies). DNA bend angles were estimated from the equation,

$$2\left\lfloor sin^{-1}\left(\frac{r/2}{d}\right)\right\rfloor ,$$ (3)

Where r is the inter-fluorophore distance as calculated by in-gel FRET and d is the inter-fluorophore distance estimated from a model of the free DNA in its canonical B-form with the fluorophores attached via 6C linkers.
