# Correction: DNA Sequence Determinants Controlling Affinity, Stability and Shape of DNA Complexes Bound by the Nucleoid Protein Fis

**DOI:** 10.1371/journal.pone.0157224

**Published:** 2016-06-21

**Authors:** The PLOS ONE Staff

There is an error in Tables 2, 3 and 4. The numbering in the first row of the second column does not line up with the corresponding DNA base sequence below. For example, in the sequence aaatttGTTTGAATTTTGAGCaaattt, the fourth capital “T” should be directly below the number 0. The publisher apologizes for the errors.

**Table 2:** Effects of flanking DNA substitutions on Fis-DNA binding affinity and complex stability^1^

			Fold-	
	-10 -9 -8 -7 -6 -5 -4 -3 -2 -1 0 1 2 3 4 5 6 7 8 9 10	K_d_ (nM)	difference^2^	t_1/2_ (min)
F1(±8T)	aaatttGTTTGAATTTTGAGCaaattt	0.2 ± 0.07	1	41 ± 4
F1±8A	aaatt**a**GTTTGAATTTTGAGC**t**aattt	2.1 ± 0.2	10	5 ± 0.5
F1±8C	aaatt**c**GTTTGAATTTTGAGC**g**aattt	0.7 ± 0.2	3	14 ± 1
F1±8G	aaatt**g**GTTTGAATTTTGAGC**c**aattt	30 ± 6	150	< 0.25
F1±9A	aaat**a**tGTTTGAATTTTGAGCa**t**attt	0.5 ± 0.2	3	22 ± 2
F1±9C	aaat**c**tGTTTGAATTTTGAGCa**g**attt	0.3 ± 0.1	2	30 ± 2
F1±9G	aaat**g**tGTTTGAATTTTGAGCa**c**attt	0.6 ± 0.2	3	25 ± 2
F1±10A	aaa**a**ttGTTTGAATTTTGAGCaa**t**ttt	0.5 ± 0.1	3	36 ± 5
F1±10C	aaa**c**ttGTTTGAATTTTGAGCaa**g**ttt	0.3 ± 0.1	2	44 ± 1
F1±10G	aaa**g**ttGTTTGAATTTTGAGCaa**c**ttt	0.5 ± 0.1	3	38 ± 5
F14	**ggg**tttGTTTGAATTTTGAGCaaa**ccc**	0.5 ± 0.1	3	ND^3^
F15	**ccc**tttGTTTGAATTTTGAGCaaa**ggg**	0.4 ± 0.2	2	ND
F16	**gcgg**ttGTTTGAATTTTGAGCaa**ccgc**	0.5 ± 0.2	3	ND
F33	aaa**gg**tGTTTGAATTTTGAGCa**cc**ttt	0.6 ± 0.1	6	ND
F34	aa**gc**ttGTTTGAATTTTGAGCaa**cg**tt	0.2 ± 0.1	1	8 ± 1
INV	**tttaaa**GTTTGAATTTTGAGC**tttaaa**	33 ± 3	165	< 0.25
INV±8G	**tttaag**GTTTGAATTTTGAGC**cttaaa**	250 ± 20	1250	ND
INV±8C	**tttaac**GTTTGAATTTTGAGC**gttaaa**	6.0 ± 2	30	ND
INV±8T	**tttaa**tGTTTGAATTTTGAGCa**ttaaa**	2.3 ± 0.7	12	4 ± 0.7
INV-CAT	**tttca**tGTTTGAATTTTGAGCa**tgaaa**	2.9 ± 1.0	15	ND
INV-GAT	**tttga**tGTTTGAATTTTGAGCa**tcaaa**	2.0 ± 0.6	10	ND
INV±9-10T	**ttta**ttGTTTGAATTTTGAGCaa**taaa**	0.4 ± 0.1	2	ND

^1^Upper case letters represent the 15 bp core Fis binding site sequence and those in lower case represent flanking DNA. Underlined and bold nucleotides highlight those that differ from F1.

^2^Fold-difference relative to the apparent equilibrium dissociation constant (K_d_) for WT Fis with F1 DNA.

^3^Not determined.

**Table 3:** Interplay between Fis residues contacting the flanking sequences and binding site variants

	-10 -9 -8 -7 -6 -5 -4 -3 -2 -1 0 1 2 3 4 5 6 7 8 9 10	Fis protein	K_d_ (nM)	Fold-difference^1^
**F1**	aaatttGTTTGAATTTTGAGCaaattt	WT	0.2 ± 0.05	1
		R71A	0.5 ± 0.2	2.5
		T75A	0.3 ± 0.8	1.5
		N73A	29 ± 0.2	140
**F27**	aaatttGTTTGAA**C**TTTGAGCaaattt	WT	0.2 ± 0.1	1
		R71A	2.8 ± 0.5	14
		T75A	3.5 ± 0.9	18
**F28**	aaatttGTTTGA**GCG**TTGAGCaaattt	WT	28 ± 4	140
		R71A	470 ± 100	2300
		T75A	> 1000	> 5000
**F32**	aaatttG**GAG**GAATTTT**CTC**Caaattt	WT	28 ± 5	140
		R71A	73 ± 11	370
		T75A	76 ± 10	380
**F1±8G**	aaatt**g**GTTTGAATTTTGAGC**c**aattt	WT	30 ± 0.8	150
		R71A	450 ± 20	2270
		T75A	48 ± 5	240

^1^Fold-difference relative to the apparent equilibrium dissociation constant (K_d_) for WT Fis with F1 DNA.

**Table 4:** Effects of flanking and core substitutions on Fis-induced DNA bending

		Gel mobility assay		In-gel FRET assay			
		(Bend angle°)		(FRET efficiency)			
		Free	Complex	Free	Complex	Distance (Å)	Angle (°)
	-10 -9 -8 -7 -6 -5 -4 -3 -2 -1 0 1 2 3 4 5 6 7 8 9 10					(complex)^1^	(complex)^2^
F1	aaatttGTTTGAATTTTGAGCaaattt	51 ± 3	119 ± 3	0.09 ± 0.01	0.27 ± 0.01	82.6	68
F1±8A	aaatt**a**GTTTGAATTTTGAGC**t**aattt	48 ± 3	109 ± 3	-	-	-	-
F1±8C	aaatt**c**GTTTGAATTTTGAGC**g**aattt	47 ± 3	126 ± 2	0.13 ± 0.01	0.26 ± 0.01	83.3	66
F1±8G	aaatt**g**GTTTGAATTTTGAGC**c**aattt	41 ± 1	99 ± 1	0.09 ± 0.01	0.23 ± 0.01	85.6	61
F1±9A	aaat**a**tGTTTGAATTTTGAGCa**t**attt	46 ± 3	104 ± 2	-	-	-	-
F1±9C	aaat**c**tGTTTGAATTTTGAGCa**g**attt	45 ± 1	99 ± 1	-	-	-	-
F1±9G	aaat**g**tGTTTGAATTTTGAGCa**c**attt	42 ± 2	89 ± 2	0.08 ± 0.03	0.18 ± 0.04	90.1	50
INV	**tttaaa**GTTTGAATTTTGAGC**tttaaa**	≤ 36	62 ± 1	0.06 ± 0.04	0.14 ± 0.01	94.7	36
F34	aa**gc**ttGTTTGAATTTTGAGCaa**cg**tt	~ 0	62 ± 3	0.12 ± 0.02	0.26 ± 0.02	83.3	66
F18	aaatttGTT**G**GAATTTT**C**AGCaaattt	51 ± 2	116 ± 1	-	-	-	-
F31	aaatttGT**AG**GAATTTT**CT**GCaaattt	51 ± 2	108 ± 2	-	-	-	-
F32	aaatttG**GAG**GAATTTT**CTC**Caaattt	52 ± 1	106 ± 2	-	-	-	-

^1^Inter-fluorophore distance calculated from FRET efficiency as detailed in the Methods.

^2^Angle calculated assuming a single central bend in the Fis-bound DNA as detailed in the Methods.
